# Supplementary material for: Composite SMG5-SMG6 PIN domain formation is essential for NMD
Source: Nat Commun. 2026 Feb 19;17:1934. doi: 10.1038/s41467-026-69819-w (PMC12923823; doi:10.1038/s41467-026-69819-w)
Supplement: Supplementary file 1 — Supplementary Information [file 41467_2026_69819_MOESM1_ESM.pdf]

SUPPLEMENTARY INFORMATION

Composite SMG5-SMG6 PIN domain formation is essential for NMD

Katharina Kurscheidt[1,#], Sophie Theunissen[2,3,#], Natalia Pasquali[4], Kerstin Becker[4], Volker Boehm[2,3], Elena Conti[1,*], Niels H. Gehring[2,3,*]

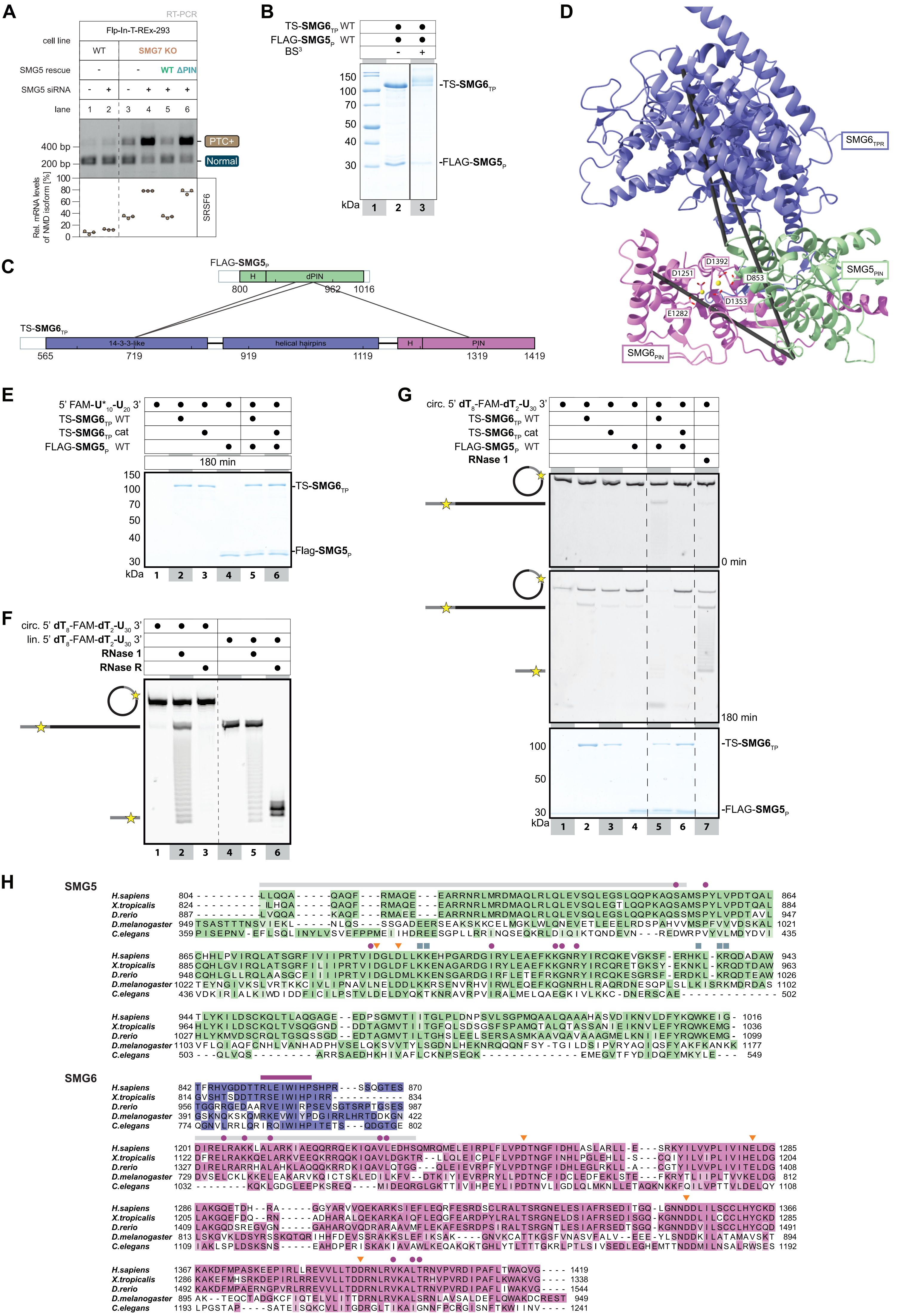

A
RT-PCR
cell line
Flp-In-T-REx-293
WT
SMG7 KO
SMG5 rescue
WT ΔPIN
SMG5 siRNA
lane
1 2 3 4 5 6
400 bp
200 bp
PTC+
Normal
Rel. mRNA levels of NMD isoform [%]
SRSF6
B
TS-SMG6TP WT
FLAG-SMG5P WT
BS3
150
100
70
50
40
30
kDa
-TS-SMG6TP
-FLAG-SMG5P
1 2 3
C
FLAG-SMG5P
H
dPIN
800
962
1016
TS-SMG6TP
14-3-3-like
helical hairpins
H
PIN
565
719
919
1119
1319
1419
D
SMG6TPR
SMG5PIN
SMG6PIN
D1392
D1251
D853
D1353
E1282
E
5' FAM-U*10-U20 3'
TS-SMG6TP WT
TS-SMG6TP cat
FLAG-SMG5P WT
180 min
-TS-SMG6TP
-Flag-SMG5P
F
circ. 5' dT8-FAM-dT2-U30 3'
lin. 5' dT8-FAM-dT2-U30 3'
RNase 1
RNase R
G
circ. 5' dT8-FAM-dT2-U30 3'
TS-SMG6TP WT
TS-SMG6TP cat
FLAG-SMG5P WT
RNase 1
0 min
180 min
-TS-SMG6TP
-FLAG-SMG5P
H
SMG5
H.sapiens
X.tropicalis
D.rerio
D.melanogaster
C.elegans
SMG6

Supplementary Fig. 1: The direct interaction of SMG5 and SMG6 enhances the endonucleolytic activity. (related to Fig. 1)

A. Rescue assay of SMG5 WT and ΔPIN. Endpoint RT-PCR detection of SRSF6 transcripts was carried out in triplicates. Upper bands represent NMD-sensitive transcript isoforms, whereas lower bands represent canonical transcript isoforms. Relative mRNA levels were quantified based on band intensities. Source data are provided as a source data file.

B. Coomassie-stained SDS-PAGE analysis of pre-incubated FLAG-$SMG5_P$ and TS-$SMG6_{TP}$ before (-) and after (+) crosslinking with BS^3. The crosslinked sample was subjected to crosslinking-mass spectrometry (XL-MS).

C. Intermolecular BS^3 crosslinks identified in XL-MS experiment between FLAG-$SMG5_P$ and TS-$SMG6_{TP}$ mapped onto the schematic domain representation of the two proteins. Dark grey lines indicate interlinks.

D. Intermolecular BS^3 crosslinks identified in XL-MS experiment between FLAG-$SMG5_P$ and TS-$SMG6_{TP}$ mapped onto the AlphaFold 3 model depicted in Fig. 1D. Dark grey lines indicate interlinks. In the conformation displayed in the AlphaFold 3 model, the interlinks violate the distance constraints of 26-30 Å for the BS^3 crosslinker. However, the crosslinked residues are located in regions with conformational flexibility.

E. Coomassie-stained SDS-PAGE analysis of proteins used in the *in vitro* nuclease assay with time-course set-up displayed in Fig. 1H (loading control). Source data are provided as a source data file.

F. Denaturing PAGE analysis of *in vitro* digest of a circularized DNA-RNA hybrid oligonucleotide with commercially available RNase I or RNase R. The corresponding linear DNA-RNA substrate served as control. Source data are provided as a source data file.

G. Denaturing PAGE analysis of *in vitro* nuclease assay assessing catalytic activity of TS-$SMG6_{TP}$ towards a circular substrate in absence and presence of FLAG-$SMG5_P$. Samples taken after 0 min (top) and 180 min (middle) show RNA input and the pattern of decay intermediates, respectively. Coomassie-stained SDS-PAGE analysis of proteins

used in the assay served as loading control (bottom). Source data are provided as a source data file.

H. Multiple sequence alignments of SMG5 and SMG6 homologues from different eukaryotes (run with full-length sequences) showing conservation of the PIN domains and preceding α-helices as well as of a loop in the SMG6 TPR-like domain. Conserved residues are coloured by domain affiliation as in Fig. 1, with catalytic residues and mutants, RNA-binding mutants and interaction mutants indicated by orange triangles, steel-blue squares, and purple circles, respectively. Dark grey and purple lines represent the α-helices preceding the PIN domains and the loop in the SMG6 TPR-like domain predicted to fold onto the extended segment of the SMG6 PIN domain, respectively.

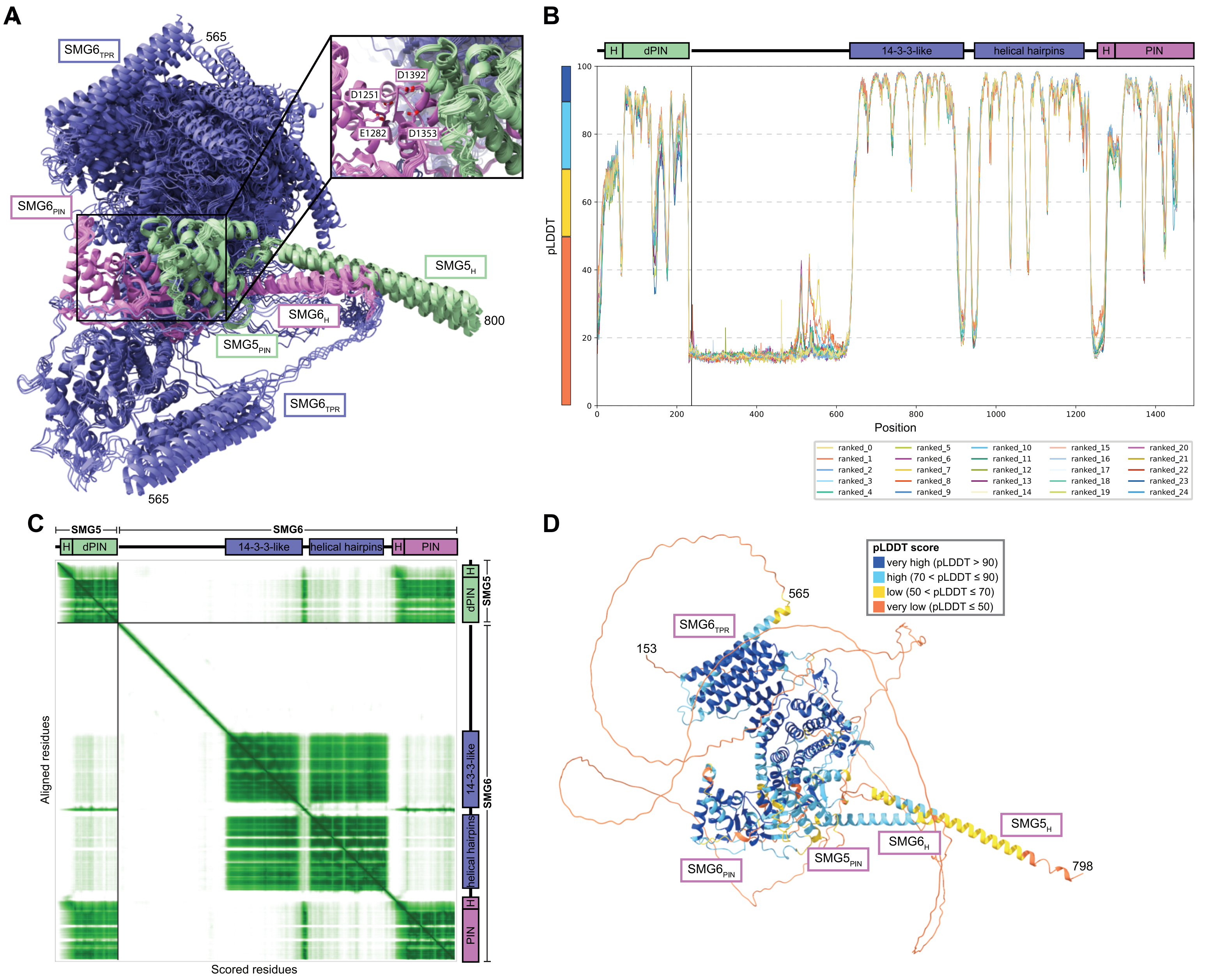

A
SMG6TPR
565
SMG6PIN
D1392
D1251
E1282
D1353
SMG5H
SMG6H
800
SMG5PIN
SMG6TPR
565
B
H
dPIN
14-3-3-like
helical hairpins
H
PIN
pLDDT
Position
ranked_0
ranked_1
ranked_2
ranked_3
ranked_4
ranked_5
ranked_6
ranked_7
ranked_8
ranked_9
ranked_10
ranked_11
ranked_12
ranked_13
ranked_14
ranked_15
ranked_16
ranked_17
ranked_18
ranked_19
ranked_20
ranked_21
ranked_22
ranked_23
ranked_24
C
SMG5
SMG6
Aligned residues
Scored residues
D
pLDDT score
very high (pLDDT > 90)
high (70 < pLDDT ≤ 90)
low (50 < pLDDT ≤ 70)
very low (pLDDT ≤ 50)
565
SMG6TPR
153
SMG5H
SMG6H
SMG5PIN
SMG6PIN
798

Supplementary Fig. 2: AlphaFold Multimer models converge on a direct interaction of SMG5 and SMG6 via their PIN domains. (related to Fig. 1)

A. Overview of AlphaFold Multimer models of an interaction of human SMG5 and SMG6 proteins via their PIN domains and close-up on the active site of the endonuclease at the interaction interface. Input sequences: $SMG5_{788\text{-}1016}$ + $SMG6_{153\text{-}1419}$. Domains are coloured as in Fig. 1 and all models are aligned on SMG6 PIN domain. SMG6 residues 153-564 are not involved in the interaction and are omitted for clarity. 25 out of 25 models are converging on extended side-by-side interaction of the PIN domains of SMG5 and SMG6 and the intramolecular **β**-sheet extension at the SMG6 PIN domain, but exhibit two diverging conformations of the SMG6 TPR-like domain. Catalytic residues of SMG6 (D1251, E1282, D1353, D1392) are indicated in the close-up.

B. Per-residue predicted local distance difference test (pLDDT) plot of the AlphaFold Multimer models for the interaction of $SMG5_{788\text{-}1016}$ + $SMG6_{153\text{-}1419}$, displayed in A.

C. Predicted aligned error (PAE) matrix of one of the AlphaFold Multimer models (ranked_0) displayed in panel A.

D. Overview of one of the AlphaFold Multimer models (ranked_0) of $SMG5_{788\text{-}1016}$ with $SMG6_{153\text{-}1419}$ coloured by pLDDT score of the C**α** atoms. Different colours correspond to the levels of confidence of the prediction along the protein sequence. Model ranked_0 is representative of one conformation of the SMG6 TPR-like domain with respect to the SMG5-SMG6 PIN-PIN interface.

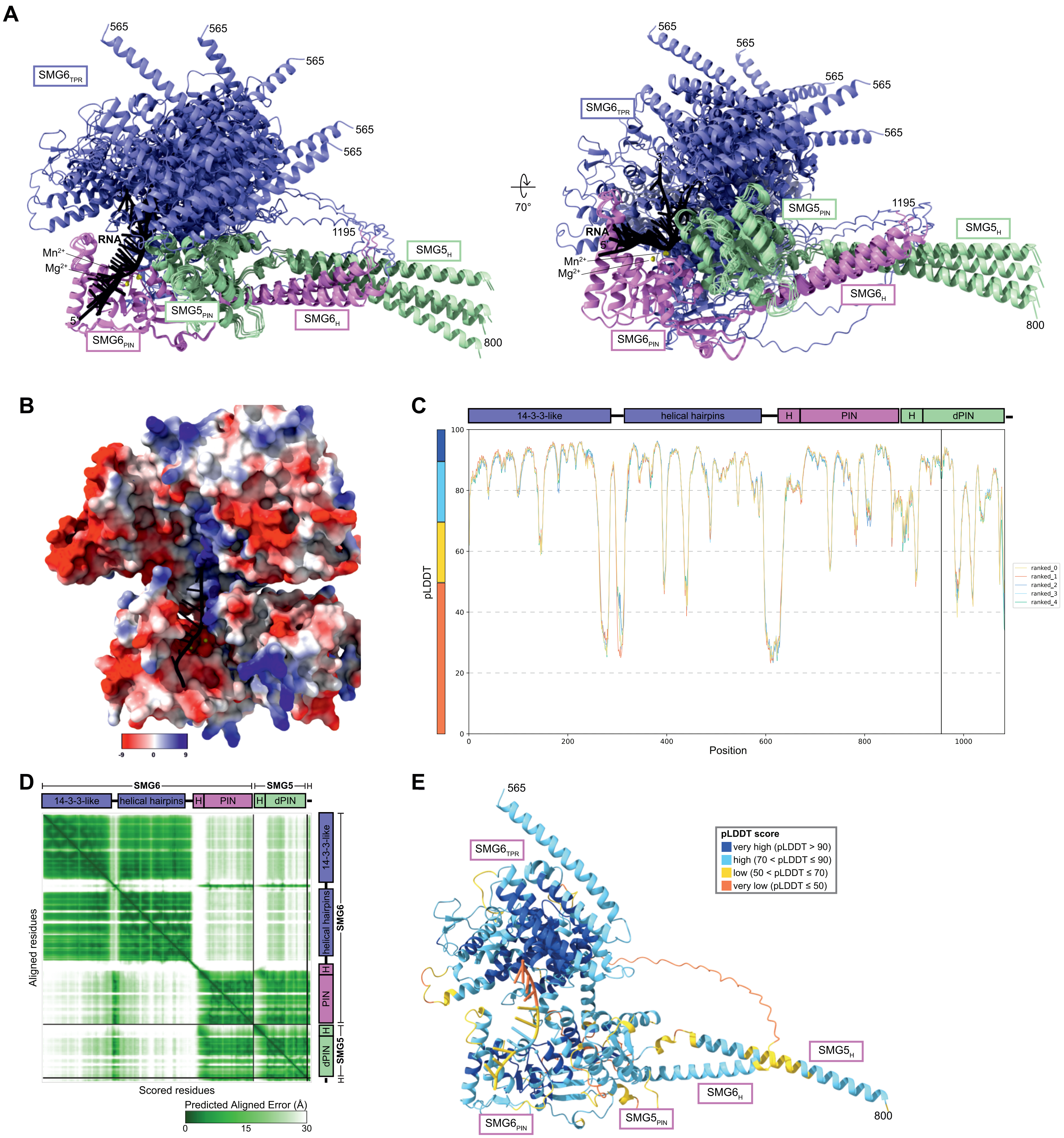
A
565
565
565
565
565
SMG6TPR
RNA
Mn2+
Mg2+
5'
3'
1195
SMG5H
SMG5PIN
SMG6H
SMG6PIN
800
70°
B
-9
0
9
C
14-3-3-like
helical hairpins
H
PIN
H
dPIN
pLDDT
Position
ranked_0
ranked_1
ranked_2
ranked_3
ranked_4
D
SMG6
SMG5
Aligned residues
Scored residues
Predicted Aligned Error (Å)
0
15
30
E
pLDDT score
very high (pLDDT > 90)
high (70 < pLDDT ≤ 90)
low (50 < pLDDT ≤ 70)
very low (pLDDT ≤ 50)

Supplementary Fig. 3: AlphaFold 3 consistently predicts the SMG5 and SMG6 PIN domains in an extended side-by-side interaction expanding the active centre. (related to Fig. 1)

A. Overview of AlphaFold 3 models of human SMG6TP and SMG5P forming an extended side-by-side arrangement via their PIN domains. Input sequences: SMG5800-1016 + SMG6565-1419 + 11-mer RNA (5'- U3-UGAAC-U3 -3') + Mn2+ + Mg2+. Two different orientations are displayed. Domains are coloured as in Fig. 1, divalent metal ions are shown as yellow spheres and RNA is shown in black. 5 out of 5 models are shown and aligned on the SMG6 PIN domain.

B. Surface representation of the AlphaFold 3 model of human SMG6TP interacting with human SMG5P as displayed in Fig. 1D. Map is coloured by electrostatic potential showing a putative RNA-binding path towards the active site of the endonuclease formed by both SMG5 and SMG6. Divalent metal ions are shown as yellow spheres and RNA is shown in black.

C. Per-residue predicted local distance difference test (pLDDT) plot of the AlphaFold 3 models for the interaction of SMG6565-1419 and SMG5800-1016 in presence of an RNA substrate and divalent metal ions displayed in A. The pLDDT scores of the Cα atoms of the protein backbone are used as estimates for the per-residue score.

D. Predicted aligned error (PAE) matrix of the AlphaFold 3 model (ranked_4) displayed in Fig. 1D.

E. Overview of the AlphaFold 3 model (ranked_4) displayed in Fig. 1D coloured by pLDDT score of the Cα atoms. Different colours correspond to the levels of confidence of the prediction.

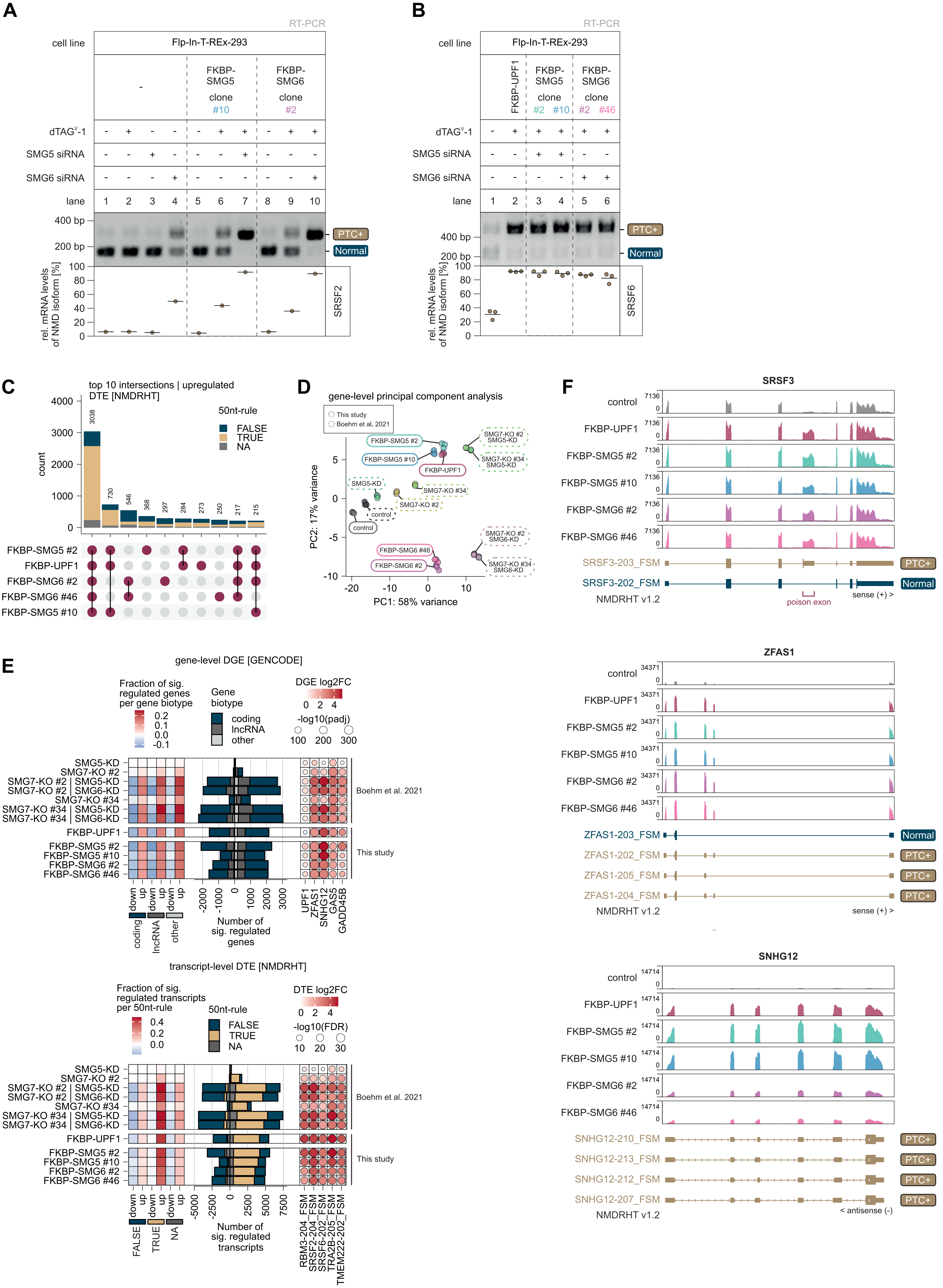

A
RT-PCR
cell line
Flp-In-T-REx-293
FKBP-SMG5 clone #10
FKBP-SMG6 clone #2
dTAG^V-1
SMG5 siRNA
SMG6 siRNA
lane
400 bp
200 bp
PTC+
Normal
rel. mRNA levels of NMD isoform [%]
SRSF2
B
RT-PCR
cell line
Flp-In-T-REx-293
FKBP-UPF1
FKBP-SMG5 clone #2 #10
FKBP-SMG6 clone #2 #46
dTAG^V-1
SMG5 siRNA
SMG6 siRNA
lane
400 bp
200 bp
PTC+
Normal
rel. mRNA levels of NMD isoform [%]
SRSF6
C
top 10 intersections | upregulated DTE [NMDRHT]
count
50nt-rule
FALSE
TRUE
NA
3038
730
546
368
297
284
273
250
217
215
FKBP-SMG5 #2
FKBP-UPF1
FKBP-SMG6 #2
FKBP-SMG6 #46
FKBP-SMG5 #10
D
gene-level principal component analysis
This study
Boehm et al. 2021
FKBP-SMG5 #2
FKBP-SMG5 #10
FKBP-UPF1
SMG7-KO #2 SMG5-KD
SMG7-KO #34 SMG5-KD
SMG5-KD
SMG7-KO #34
SMG7-KO #2
control
SMG7-KO #2 SMG6-KD
SMG7-KO #34 SMG6-KD
FKBP-SMG6 #46
FKBP-SMG6 #2
PC1: 58% variance
PC2: 17% variance
E
gene-level DGE [GENCODE]
Fraction of sig. regulated genes per gene biotype
Gene biotype
coding
lncRNA
other
DGE log2FC
-log10(padj)
SMG5-KD
SMG7-KO #2
SMG7-KO #2 | SMG5-KD
SMG7-KO #2 | SMG6-KD
SMG7-KO #34
SMG7-KO #34 | SMG5-KD
SMG7-KO #34 | SMG6-KD
FKBP-UPF1
FKBP-SMG5 #2
FKBP-SMG5 #10
FKBP-SMG6 #2
FKBP-SMG6 #46
Boehm et al. 2021
This study
Number of sig. regulated genes
UPF1
ZFAS1
SNHG12
GAS5
GADD45B
transcript-level DTE [NMDRHT]
Fraction of sig. regulated transcripts per 50nt-rule
50nt-rule
FALSE
TRUE
NA
DTE log2FC
-log10(FDR)
Number of sig. regulated transcripts
RBM3-204_FSM
SRSF2-204_FSM
SRSF6-202_FSM
TRA2B-205_FSM
TMEM222-202_FSM
F
SRSF3
control
FKBP-UPF1
FKBP-SMG5 #2
FKBP-SMG5 #10
FKBP-SMG6 #2
FKBP-SMG6 #46
SRSF3-203_FSM
SRSF3-202_FSM
poison exon
NMDRHT v1.2
sense (+) >
ZFAS1
ZFAS1-203_FSM
ZFAS1-202_FSM
ZFAS1-205_FSM
ZFAS1-204_FSM
SNHG12
SNHG12-210_FSM
SNHG12-213_FSM
SNHG12-212_FSM
SNHG12-207_FSM
< antisense (-)

Supplementary Fig. 4: RNA-seq data of SMG5/SMG6-depleted cells revealed significant changes on gene- and transcript level. (related to Fig. 2)

A. Endpoint RT-PCR detection of SRSF2 transcript isoforms was carried out in the indicated Flp-In-T-REx-293 degron cell lines with either no treatment, treatment with 250 µM dTAG^{V}-1 or the combination of dTAG^{V}-1 and the respective knockdown. Upper bands represent NMD-sensitive transcript isoforms, whereas lower bands represent canonical transcript isoforms. Relative mRNA levels were quantified based on their band intensity (n = 3 biologically independent samples). Source data are provided as a source data file.

B. Endpoint RT-PCR detection of SRSF6 transcript isoforms was carried out in triplicates. Upper bands represent NMD-sensitive transcript isoforms, whereas lower bands represent canonical transcript isoforms. Source data are provided as a source data file.

C. UpSet plot of significantly upregulated NMDRHT-annotated transcripts, stratified by predicted NMD status (50-nucleotide rule). Only the top 10 intersections are shown.

D. Gene-level principal component analysis (PCA) of transcriptome profiles from RNA-seq data generated in this study compared with various SMG5/6/7 depletion data from Boehm *et al.* 2021.

E. Comparison of published SMG5/6/7 depletion data from Boehm *et al.* 2021 with FKBP-UPF1/SMG5/SMG6 degron cell lines and control conditions regarding significantly regulated genes stratified by GENCODE biotype (top) and significantly regulated NMDRHT-annotated transcripts (bottom). The heatmap (left) depicts the fraction of genes per gene biotype or transcripts per 50-nt rule that was significantly up- or downregulated, in relation to total expressed genes or transcript of the respective type. The bar plot (middle) depicts the absolute number of up- or downregulated genes or transcripts per gene biotype or 50-nt rule. The dot plot (right) highlights the expression changes (colour) and statistical significance (point size) for individual genes or transcripts. Differential gene expression (DGE) and differential transcript expression (DTE) are depicted as log2 fold changes (log2FC).

F. Read coverage of SRSF3, ZFAS1 and SNHG12 from RNA-seq data of control and FKBP-

UPF1/SMG5/SMG6 degron cell lines are shown as Integrative Genomics Biewer (IGV) snapshots. The NMDRHT-annotated isoforms and their predicted NMD status are schematically indicated below.

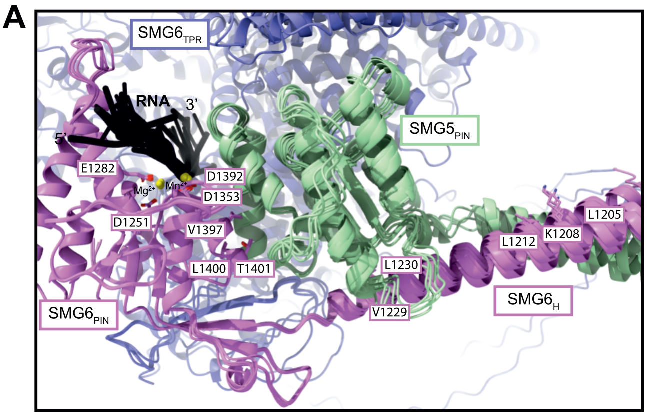

SMG6 mutants	
D1353A	C1
D1392A	C2
Δ845-868	ΔL
L1205A K1208D L1212D	I1
V1229E L1230E	I2
V1397R L1400R T1401R	I3

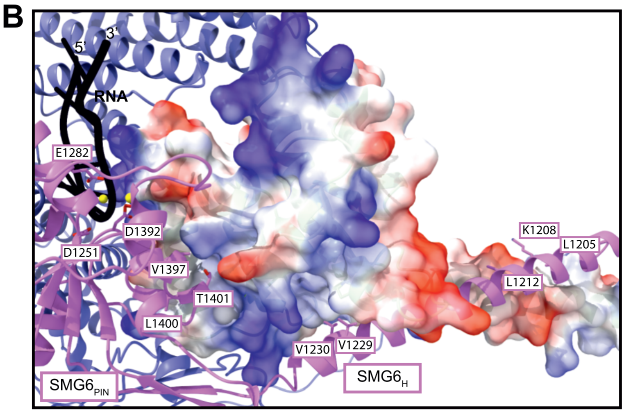

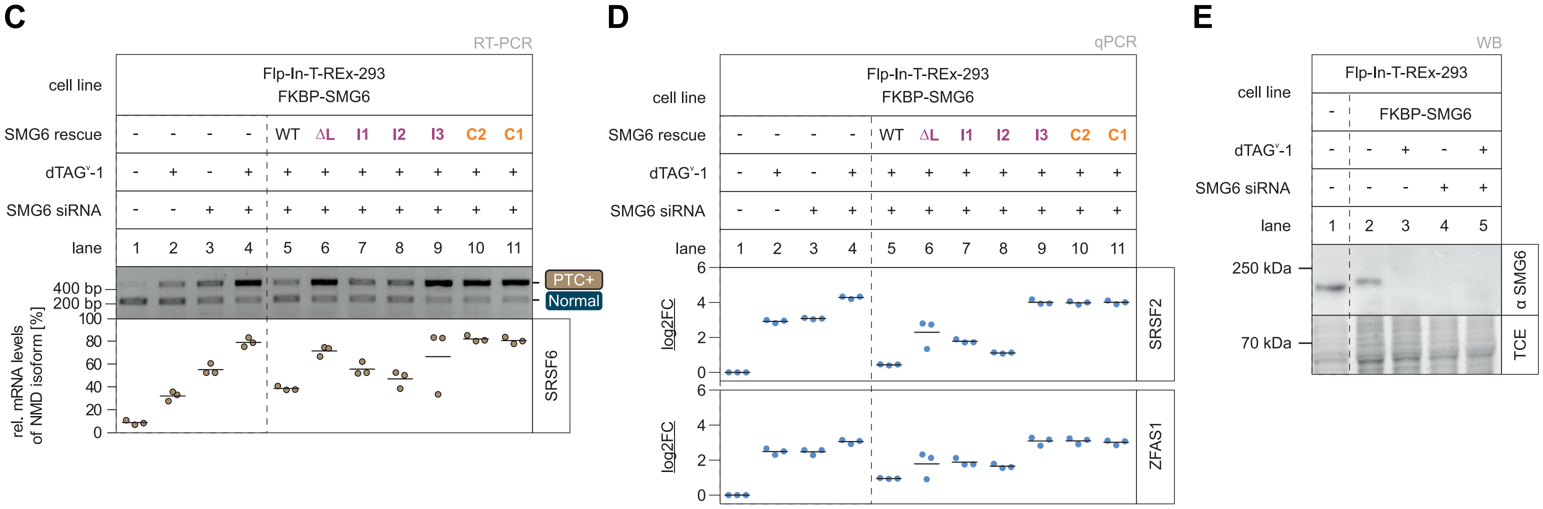

Supplementary Fig. 5: SMG6 mutants impair NMD in human cells. (related to Fig. 3)

A. Close-up on the extended active site and PIN-PIN interface formed by SMG5-SMG6 as predicted by AlphaFold 3 and displayed in Fig. 1D. 5 out of 5 models are shown. Domains are coloured as in Fig. 1, divalent metal ions are shown as yellow spheres, and RNA is shown in black. Catalytic residues (D1251, E1282, D1353, D1392) and mutated residues of SMG6 are labelled and displayed as sticks. Mutants of SMG6 putatively impairing catalysis (Cx; orange) or disrupting protein-protein interaction (Ix; purple) and used in this study are summarised in the table (right).

B. Close-up on the extended active site and the PIN-PIN interface formed by SMG5-SMG6 as predicted by AlphaFold 3 with SMG5 shown in surface representation. Domains of SMG6 are coloured as in Fig. 1, while the surface of SMG5 is coloured according to electrostatic potential. Catalytic residues (D1251, E1282, D1353, D1392) and mutated residues of SMG6 are labelled and displayed as sticks.

C. Rescue assay of SMG6 mutants in SMG6 degron cells. Endpoint RT-PCR detection of SRSF6 transcript isoforms of SMG6 mutants was carried out in triplicates. Upper bands represent NMD-sensitive transcript isoforms, whereas lower bands represent canonical transcript isoforms. Relative mRNA levels of the NMD-sensitive isoforms were quantified based on their band intensity. Source data are provided as a source data file.

D. Rescue assay of SMG6 mutants in SMG6 degron cells. Probe-based qPCR detection of SRSF2 and ZFAS1 transcript isoforms. The ratios of SRSF2 to the B2M reference, and ZFAS1 to the B2M reference were calculated; data points and means from the qPCRs are plotted as log2 fold change (log2FC) (n=3 biologically independent samples). Source data are provided as a source data file.

E. Western blot analysis using an anti-SMG6 antibody revealed successful knockdown and $dTAG^{V}$-1 mediated degradation of endogenous SMG6. TCE staining served as loading control. Source data are provided as a source data file.

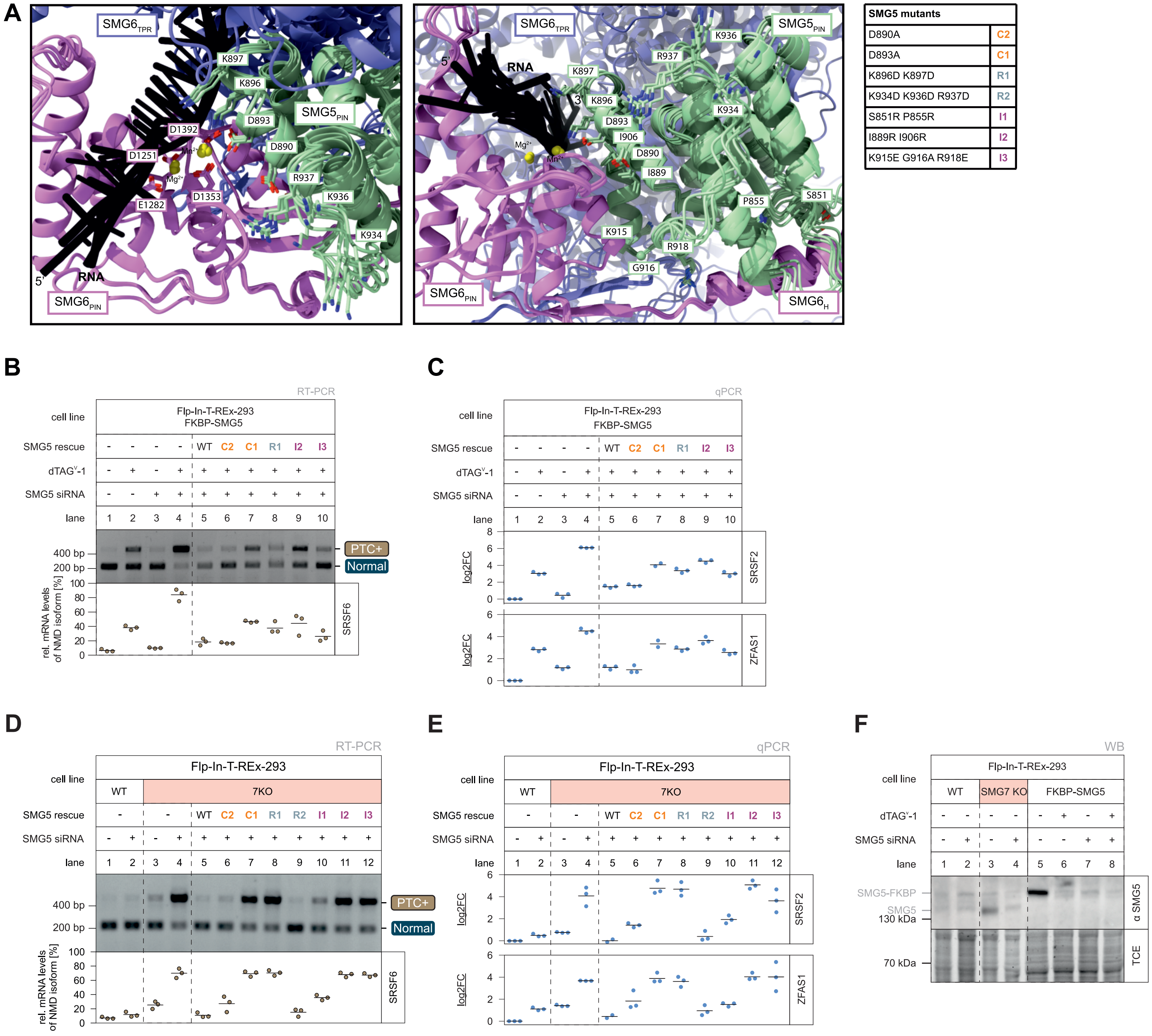
A
SMG6TPR
SMG5PIN
SMG6PIN
SMG6H
RNA
5'
3'
K897
K896
D893
D890
D1392
D1251
E1282
D1353
R937
K936
K934
I906
I889
P855
S851
K915
R918
G916
Mg2+
Mn2+
SMG5 mutants
D890A C2
D893A C1
K896D K897D R1
K934D K936D R937D R2
S851R P855R I1
I889R I906R I2
K915E G916A R918E I3
B
RT-PCR
cell line
Flp-In-T-REx-293 FKBP-SMG5
SMG5 rescue
dTAGV-1
SMG5 siRNA
lane
400 bp
200 bp
PTC+
Normal
rel. mRNA levels of NMD isoform [%]
SRSF6
C
qPCR
log2FC
SRSF2
ZFAS1
D
Flp-In-T-REx-293
WT
7KO
E
F
WB
SMG7 KO
FKBP-SMG5
SMG5-FKBP
SMG5
130 kDa
70 kDa
α SMG5
TCE

Supplementary Fig. 6: SMG5 mutants impair SMG6 catalysis. (related to Fig. 4)

A. Close-up on the extended active site and the PIN-PIN interface formed by SMG5-SMG6 as predicted by AlphaFold 3 and displayed in Fig. 1D. 5 out of 5 models are shown in two different orientations. Domains are coloured as in Fig. 1, divalent metal ions are shown as yellow spheres, RNA is shown in black. Catalytic residues of SMG6 and mutated residues of SMG5 are labelled and displayed as sticks. Mutants of SMG5 putatively impairing catalysis (Cx; orange), RNA-binding (Rx; steel-blue) or protein-protein interaction (Ix; purple) are summarised in the table (right).

B. Rescue assay of SMG5 mutants in SMG5 degron cells. Endpoint RT-PCR detection of SRSF6 transcripts was carried out in triplicates. Upper bands represent NMD-sensitive transcript isoforms, whereas lower bands represent canonical transcript isoforms. Indicated fragment sizes (bp) are estimated. Relative mRNA levels were quantified based on their band intensity. Source data are provided as a source data file.

C. Probe-based qPCR of SMG5 mutants in SMG5 degron cells used in B. Analysis was conducted to calculate the ratios of SRSF2 and ZFAS1 relative to the B2M reference; data points and means from the qPCRs are plotted as log2 fold change (log2FC) (n=3 biologically independent samples). Source data are provided as a source data file.

D. Rescue assay of SMG5 mutants in SMG7 KO cells. Endpoint RT-PCR detection of SRSF6 transcripts was carried out in triplicates. Upper bands represent NMD-sensitive transcript isoforms, whereas lower bands represent canonical transcript isoforms. Relative mRNA levels were quantified based on their band intensity. Source data are provided as a source data file.

E. Probe -based qPCR of SMG5 mutants in SMG7 KO cells used in D. The ratios of SRSF2 and ZFAS1 transcript isoforms were calculated to the B2M reference; data points and means from the qPCRs are plotted as log2 fold change (log2FC) (n=3 biologically independent samples). Source data are provided as a source data file.

F. Western Blot analysis of WT, SMG7 KO and FKBP-SMG5 cells using an anti-SMG5 antibody revealed successful knockdown and $dTAG^V$-1-mediated degradation of endogenous SMG5. TCE staining served as loading control. Source data are provided as a source data file.
