# Supplementary material for: Composite SMG5-SMG6 PIN domain formation is essential for NMD
Source: Nat Commun. 2026 Feb 19;17:1934. doi: 10.1038/s41467-026-69819-w (PMC12923823; doi:10.1038/s41467-026-69819-w)
Supplement: Supplementary file 2 — Description of Additional Supplementary Files [file 41467_2026_69819_MOESM2_ESM.pdf]

Description of Additional Supplementary Files

Supplementary Data 1: List of datasets, cell lines, bacteria, siRNAs, primers, plasmids, antibodies, RNAs, peptides and enzymes used in this study.

Supplementary Data 2: Scoring metrics of AlphaFold runs.
