# Supplementary material for: Composite SMG5-SMG6 PIN domain formation is essential for NMD
Source: Nat Commun. 2026 Feb 19;17:1934. doi: 10.1038/s41467-026-69819-w (PMC12923823; doi:10.1038/s41467-026-69819-w)
Supplement: Supplementary file 5 — Reporting Summary [file 41467_2026_69819_MOESM5_ESM.pdf]

nature portfolio

Corresponding author(s): Elena Conti & Niels H. Gehring

Last updated by author(s): Jan 28, 2026

Reporting Summary

Nature Portfolio wishes to improve the reproducibility of the work that we publish. This form provides structure for consistency and transparency in reporting. For further information on Nature Portfolio policies, see our Editorial Policies and the Editorial Policy Checklist.

Statistics

For all statistical analyses, confirm that the following items are present in the figure legend, table legend, main text, or Methods section.

n/a	Confirmed	
☐	☒	The exact sample size (*n*) for each experimental group/condition, given as a discrete number and unit of measurement
☐	☒	A statement on whether measurements were taken from distinct samples or whether the same sample was measured repeatedly
☐	☒	The statistical test(s) used AND whether they are one- or two-sided<br>*Only common tests should be described solely by name; describe more complex techniques in the Methods section.*
☒	☐	A description of all covariates tested
☒	☐	A description of any assumptions or corrections, such as tests of normality and adjustment for multiple comparisons
☐	☒	A full description of the statistical parameters including central tendency (e.g. means) or other basic estimates (e.g. regression coefficient) AND variation (e.g. standard deviation) or associated estimates of uncertainty (e.g. confidence intervals)
☐	☒	For null hypothesis testing, the test statistic (e.g. *F*, *t*, *r*) with confidence intervals, effect sizes, degrees of freedom and *P* value noted<br>*Give P values as exact values whenever suitable.*
☒	☐	For Bayesian analysis, information on the choice of priors and Markov chain Monte Carlo settings
☒	☐	For hierarchical and complex designs, identification of the appropriate level for tests and full reporting of outcomes
☒	☐	Estimates of effect sizes (e.g. Cohen's *d*, Pearson's *r*), indicating how they were calculated

Our web collection on statistics for biologists contains articles on many of the points above.

Software and code

Policy information about availability of computer code

Data collection	Western blots were analyzed using the Fusion FX-6 Edge system (Vilber Lourmat) using the Evolution-Capt Edge software (version 18.16g). The Gel Doc XR+ (Bio-Rad) and Image Lab software (6.0.1) was used to visualize EtBr- or TCE-stained gels. The CFX96 Touch Real-Time PCR Detection System (Bio-Rad) with Bio-Rad CFX Manager software (version 3.0) was used for quantitative RT-PCR. The RNA-Seq reads were generated with an Illumina NovaSeq6000 sequencing instrument and a PE100 protocol. Details for RNA-Seq data collection can be found in the Methods section. Structure predictions were obtained using AlphaFold Multimer v2.3 and AlphaFold 3. Proteome Discoverer v2.5.0.400 was used to process the raw data of the crosslinking MS experiment.
Data analysis	RNA-seq reads were aligned against the human genome (version 38, GENCODE release 42 transcript annotations supplemented with SIRVomeERCCome annotations from Lexogen) using the STAR read aligner (version 2.7.10b). Transcript abundance estimates were computed with Salmon (version 1.9.0) with a decoy-aware transcriptome. Differential gene expression analysis was performed with the DESeq2 R package (version 1.40.1). Differential transcript expression analysis was performed with the edgeR R package (version 3.42.4). Further details for RNA-Seq data analysis can be found in the Methods section.<br>RNA gels of in vitro activity assays were analyzed using ImageJ2 (version 2.14.0/1.54f) ; ChimeraX (versions 1.9 and 1.10) was used for analysis of AlphaFold Multimer and AlphaFold 3 models and visualization of crosslinking MS data. Crosslinking MS data were analzyed using the xiNET tool (https://crosslinkviewer.org/index.php) as well as the XMAS extension for ChimeraX.

For manuscripts utilizing custom algorithms or software that are central to the research but not yet described in published literature, software must be made available to editors and reviewers. We strongly encourage code deposition in a community repository (e.g. GitHub). See the Nature Portfolio guidelines for submitting code & software for further information.

Data

Policy information about availability of data

All manuscripts must include a data availability statement. This statement should provide the following information, where applicable:

- Accession codes, unique identifiers, or web links for publicly available datasets
- A description of any restrictions on data availability
- For clinical datasets or third party data, please ensure that the statement adheres to our policy

Sequencing data generated in this study have been deposited at BioStudies/ArrayExpress under accession number E-MTAB-15521 [https://www.ebi.ac.uk/biostudies/ArrayExpress/studies/E-MTAB-15521]. Crosslinking MS data have been deposited to the ProteomeXchange Consortium via the PRIDE partner repository with the dataset identifier PXD072588 [http://proteomecentral.proteomexchange.org/cgi/GetDataset?ID=PXD072588]. sed published protein structures were PRORP1 from A. thaliana (PDB: 4G24) [https://doi.org/10.2210/pdb4G24/pdb]. Raw data including original western blot, RT-PCR scans, RNA-seq analyses (including statistical parameters) and AlphaFold outputs have been deposited at Zenodo [https://doi.org/10.5281/zenodo.18401619]. Scoring metrics of AlphaFold runs are summarized in Supplementary Data 2.

Research involving human participants, their data, or biological material

Policy information about studies with human participants or human data. See also policy information about sex, gender (identity/presentation), and sexual orientation and race, ethnicity and racism.

Reporting on sex and gender	NA (this study did not involve human participants or their data)
Reporting on race, ethnicity, or other socially relevant groupings	NA (this study did not involve human participants or their data)
Population characteristics	NA (this study did not involve human participants or their data)
Recruitment	NA (this study did not involve human participants or their data)
Ethics oversight	NA (this study did not involve human participants or their data)

Note that full information on the approval of the study protocol must also be provided in the manuscript.

Field-specific reporting

Please select the one below that is the best fit for your research. If you are not sure, read the appropriate sections before making your selection.

☒ Life sciences ☐ Behavioural & social sciences ☐ Ecological, evolutionary & environmental sciences

For a reference copy of the document with all sections, see nature.com/documents/nr-reporting-summary-flat.pdf

Life sciences study design

All studies must disclose on these points even when the disclosure is negative.

Sample size	No statistical methods were performed to predetermine sample size. The numbers of replicates (=sample size) are given in the Methods or Figure Legend for each experiment. For most experiments this sample size was three independent biological replicates, as is standard for similar molecular biology experiments. Comparable sample sizes were chosen that allow data reproducibility for each experimental conditions. For all data - if possible - positive and negative controls were included.
Data exclusions	No data exclusion criteria were pre-established besides default significance and effect cutoffs in analyses such as RNA-sequencing (values described in the manuscript), which are according to standards in the field. These default cutoffs were: Differential gene expression |log2FoldChange|> 1 and adjusted p-value (padj) < 0.0001; Differential transcript expression |log2FoldChange| > 1 and adjusted p-value (FDR) < 0.0001
Replication	RNA-sequencing runs were performed once with triplicates of distinct samples for each condition.<br>Quantified end-point PCR and qPCR experiments were performed at least with triplicates of distinct samples for each condition. All attempts at replication were successful.
Randomization	The samples were not randomized in this study, since we did not perform experiments or statistical analyses that require randomization.
Blinding	The investigators were not blinded during data collection because the experiments did not require blinding and were readily controlled without blinding. Investigators were not blinded during data analyses because the key findings are supported by quantitative measurements (with statistical testing where relevant) that do not rely heavily on subjective judgment for interpretation.

Reporting for specific materials, systems and methods

We require information from authors about some types of materials, experimental systems and methods used in many studies. Here, indicate whether each material, system or method listed is relevant to your study. If you are not sure if a list item applies to your research, read the appropriate section before selecting a response.

Materials & experimental systems

n/a	Involved in the study
☐	☒ Antibodies
☐	☒ Eukaryotic cell lines
☒	☐ Palaeontology and archaeology
☒	☐ Animals and other organisms
☒	☐ Clinical data
☒	☐ Dual use research of concern
☒	☐ Plants

Methods

n/a	Involved in the study
☒	☐ ChIP-seq
☒	☐ Flow cytometry
☒	☐ MRI-based neuroimaging

Antibodies

Antibodies used

Description in the following order: Name | Dilution | Source
Rabbit polyclonal anti-SMG5 | 1:1000 | Proteintech Cat# 12694-1-AP, RRID:AB_2270781
Rabbit polyclonal anti-SMG6 | 1:3000 | Abcam Cat# ab87539, RRID:AB_10674461
Goat polyclonal anti-UPF1/RENT1 | 1:2000 | Bethyl Cat# A300-036A, RRID:AB_203272
Rabbit polyclonal anti-SMG7 | 1:1000 | Biorbyt Cat# orb214942, RRID:NA
Mouse monoclonal anti-FLAG M2 | 1:3000 Sigma-Aldrich | Cat# F3165, RRID:AB_259529
Goat polyclonal anti-V5 | 1:5000 Bethyl | Cat#A190-119A, RRID:AB_67317
Multi-rAb HRP-Goat Anti-Mouse Recombinant Secondary Antibody (H+L) | 1:5000 | Proteintech Cat# RGAM001, RRID:AB_3068333
Multi-rAb HRP-Goat Anti-Rabbit Recombinant Secondary Antibody (H+L) | 1:6000 | Proteintech Cat# RGAR001, RRID:AB_3073505
Peroxidase-AffiniPure Donkey anti-Goat IgG | 1:5000 | Jackson ImmunoResearch Labs Cat# 705-035-147, RRID:AB_2313587

Validation

All primary antibodies were recommended or validated by the manufacturer for western blotting and have been tested with suitable overexpression, knockdown, or knockout samples.
Validation statements from the manufacturer (datasheet or homepage):
Rabbit polyclonal anti-SMG5; Proteintech Cat# 12694-1-AP; application(s): WB, IHC, IF/ICC, ELISA; Reactivity: Human, Mouse
Rabbit polyclonal anti-SMG6; Abcam Cat# ab87539; application(s): WB, ICC/IF; Reactivity: Human, Mouse
Goat polyclonal anti-UPF1/RENT1; Bethyl Cat# A300-036A; application(s): WB; Reactivity: Mouse, Human
Rabbit polyclonal anti-SMG7; Biorbyt Cat# orb214942; application(s): ICC, IF, IHC-P, IP, WB; Reactivity: Human
Mouse monoclonal anti-FLAG M2; Cat# F3165; application(s): western blot; Reactivity: all
Goat polyclonal anti-V5; Cat#A190-119A; application(s): ELISA, ICC, IP, WB; Reactivity: all
Multi-rAb HRP-Goat Anti-Mouse Recombinant Secondary Antibody (H+L); Proteintech Cat# RGAM001; application(s): ELISA, WB, Dot blot; Reactivity: Mouse
Multi-rAb HRP-Goat Anti-Rabbit Recombinant Secondary Antibody (H+L); Proteintech Cat# RGAR001; application(s): ELISA, WB, Dot blot; Reactivity: Rabbit
Peroxidase-AffiniPure Donkey anti-Goat IgG; Jackson ImmunoResearch Labs Cat# 705-035-147; application(s): ELISA, WB, IHC; Reactivity: Goat

Eukaryotic cell lines

Policy information about cell lines and Sex and Gender in Research

Cell line source(s)

Parental Flp-In-T-REx-293 cells were purchased from Thermo Fisher Scientific; Cat# R78007; RRID:CVCL_U427

Authentication

The parental Flp-In-T-REx-293 cell line was not further authenticated. CRISPR/Cas-edited cell lines were authenticated by western blotting, PCR of cDNA and/or genomic DNA, and in some cases by RNA-sequencing. Generated stable cell lines were authenticated by western blotting to detect the expressed protein.

Mycoplasma contamination

The parental cell lines was routinely tested and was free of mycoplasma contamination. Subsequent cell lines were not re-tested during the course of this study.

Commonly misidentified lines
(See ICLAC register)

No commonly misidentified cell lines were used in the study.

Plants

Seed stocks	NA (No plants were used in this study)
Novel plant genotypes	NA (No plants were used in this study)
Authentication	NA (No plants were used in this study)
